# Development of deep learning models for high-resolution exposome mapping and health impact assessment

**DOI:** 10.3389/fpubh.2025.1565471

**Published:** 2025-06-04

**Authors:** Lan Luan, Zhu Daoyu

**Affiliations:** ^1^College of Computer and Information Engineering, Guizhou University of Commerce, Guizhou, China; ^2^College of Physical Education, Xinyang Normal University, Xinyang, Henan, China

**Keywords:** exposome mapping, high-resolution modeling, deep learning, multi-modal data integration, uncertainty quantification

## Abstract

**Introduction:**

The study of environmental health and the exposome is becoming increasingly vital as researchers aim to untangle the complex interactions between environmental exposures and human health outcomes. Traditional exposome mapping methods often face limitations, such as low spatial-temporal resolution, challenges in integrating multi-modal data sources, and inadequate handling of uncertainties in exposure quantification.

**Methods:**

To address these gaps, we introduce an innovative framework that leverages advanced deep learning techniques, adaptive optimization strategies, and multi-scale data integration to achieve high-resolution exposome modeling. Central to our approach is the Adaptive Multi-Scale Exposure Network (AMSEN), a hierarchical deep learning model designed to harmonize diverse data streams, such as satellite imagery, wearable sensors, and geospatial analytics, while addressing the challenges of multi-scale variability and measurement uncertainties. AMSEN incorporates cross-modal fusion mechanisms, spatiotemporal feature extraction, and uncertainty quantification. Complementing AMSEN, the Adaptive Exposure Optimization Strategy (AEOS) enhances model efficiency and accuracy through dynamic resource allocation, uncertainty-guided refinement, and domain-specific prior enforcement.

**Results:**

These methodologies significantly advance the capabilities of exposome research by providing a robust, adaptive, and high-resolution modeling framework.

**Discussion:**

The experimental findings highlight the effectiveness of our approach, showcasing enhancements in exposure prediction precision, computational performance, and practical insights for public health policymaking. This work aligns with the objectives of advancing environmental health sciences by offering novel tools for exposome quantification and health impact assessment.

## 1 Introduction

The study of the exposome, which encompasses the totality of environmental exposures an individual encounters throughout their lifetime, is essential for understanding the multifactorial causes of human diseases (1). High-resolution exposome mapping not only enables precise identification of exposure sources but also aids in quantifying their impact on health outcomes, thereby bridging the gap between environmental exposure and epidemiological studies (2). This task is complex, as it involves integrating diverse data modalities such as satellite imagery, air quality measurements, wearable sensors, and socio-demographic data (3). Traditional methods are often insufficient due to their limited ability to process heterogeneous and high-dimensional datasets, while newer approaches, including machine learning and deep learning, promise to overcome these limitations (4). The development of such models is not only critical for advancing environmental health research but also has far-reaching implications for public health policies, personalized healthcare, and urban planning (5). There is a growing need to explore how computational models can accurately characterize the exposome, assess health risks, and provide actionable insights.

Early computational efforts in exposome research focused on structuring domain-specific knowledge to support exposure assessment (6). Researchers developed formalized systems that encoded expert insights to interpret environmental indicators, enabling rule-based inferences from spatial and temporal datasets (7). These strategies facilitated initial progress in linking environmental contexts with health outcomes and proved useful for targeted exposure estimation (8). However, their rigidity and dependence on manually constructed frameworks limited their capacity to adapt to complex, large-scale, and dynamic data environments. As exposome-related datasets grew in scope and granularity, such limitations became increasingly apparent, revealing the need for more adaptive and scalable solutions (9, 10).

In response to these challenges, adaptive modeling techniques were introduced to better accommodate the expanding landscape of exposome data (11). By leveraging statistical learning frameworks, researchers began to construct predictive models capable of uncovering hidden associations between environmental indicators and health risks (12). Algorithms such as support vector machines, random forests, and gradient boosting machines enhanced the accuracy and efficiency of exposure estimates by integrating diverse inputs from sensors, imaging, and demographic databases (13). These models represented a substantial advancement, yet they often required extensive tuning and expert-guided feature design (14). Moreover, understanding the causal mechanisms behind their predictions remained difficult, prompting concerns about interpretability and trustworthiness in health-related applications (15).

Recent developments in representation learning have further advanced the field by enabling automatic extraction of relevant features from large, complex datasets (16). Neural network architectures, including convolutional and recurrent models, have demonstrated strong capabilities in processing multi-dimensional information such as geospatial images, time series, and mobility patterns (17). Emerging transformer-based frameworks and pre-trained models like BERT and GPT have also been adapted to handle multimodal exposure data, offering improved capacity to model intricate relationships among environmental and biological variables (18). These models have achieved notable performance in fine-grained exposure estimation and health outcome prediction (19). Nonetheless, challenges such as generalizability across populations, the need for large annotated datasets, and the opaque nature of model outputs continue to present barriers to their widespread adoption in exposome research (20).

Based on the limitations of prior approaches, we propose a novel methodology for high-resolution exposome mapping and health impact assessment using advanced deep learning architectures. Our method addresses the challenges of data heterogeneity, scalability, and interpretability by incorporating multi-modal data fusion techniques, self-supervised learning for data-scarce environments, and explainable AI (XAI) frameworks. By leveraging state-of-the-art models tailored for environmental health research, we aim to improve the accuracy, efficiency, and transparency of exposome analyses. This approach not only builds upon the strengths of existing methods but also mitigates their weaknesses by prioritizing robustness, adaptability, and user interpretability. In doing so, our method has the potential to redefine the landscape of exposome research and its applications in public health.

- Our approach integrates cutting-edge multi-modal data fusion techniques and self-supervised learning to handle heterogeneous and incomplete datasets effectively.
- The proposed models are designed to perform efficiently across diverse environments and data sources, ensuring wide applicability and scalability.
- Experimental results show significant improvements in the accuracy of exposome mapping and health risk predictions, offering actionable insights for public health interventions.

## 2 Related work

### 2.1 Deep learning in exposome mapping

Deep learning has been increasingly employed in exposome research, particularly for mapping complex environmental exposures (21). The exposome, which encompasses the totality of environmental exposures an individual encounters throughout their lifetime, is inherently multidimensional and dynamic (22). Capturing this complexity requires models capable of handling high-dimensional, spatiotemporal, and heterogeneous data. Deep learning models, such as convolutional neural networks (CNNs) and recurrent neural networks (RNNs), have been applied to model various components of the exposome, such as air pollution, noise levels, and green space distributions. These models are well-suited for processing large-scale environmental datasets that often come in the form of satellite imagery, geospatial data, and time-series measurements. Recent advances in satellite imaging and sensor technology have contributed to the availability of high-resolution environmental data, enabling deep learning applications in fine-scale exposome mapping (23). CNNs, for example, have been extensively used for extracting spatial features from remote sensing data. These models have demonstrated superior performance in predicting air pollution concentrations, such as PM_2.5_ and NO_2_, by integrating meteorological data, land-use features, and population density (24). Advanced architectures like U-Nets have been adopted for pixel-wise prediction tasks, such as land cover classification and vegetation analysis, providing valuable inputs for exposome studies. In addition, transfer learning has been used to leverage pre-trained models on large image datasets to improve the performance of domain-specific tasks with limited labeled data. Temporal dynamics are also critical in exposome mapping, as environmental exposures often vary over time (25) (RNNs) and long short-term memory (LSTM) networks have demonstrated their potential in modeling temporal dependencies within exposure data. These approaches have been applied to predict air quality indices and track seasonal trends in environmental pollutants. The integration of temporal and spatial information has led to the development of spatiotemporal deep learning models, which combine CNNs and RNNs to address the dynamic nature of the exposome. These hybrid architectures are particularly effective for applications requiring both fine spatial resolution and temporal continuity. Despite these advances, challenges remain in the application of deep learning to exposome mapping (26). One key issue is the lack of standardized datasets and benchmarks, which limits the comparability of model performance across studies. Another challenge is the interpretability of deep learning models, as the black-box nature of these algorithms makes it difficult to derive mechanistic insights into the relationships between environmental exposures and health outcomes. Explainable AI techniques, such as attention mechanisms and feature importance analyses, have been proposed to address this issue. The fusion of multi-modal data, such as integrating satellite imagery with wearable sensor measurements, presents computational and methodological hurdles that warrant deeper investigation.

### 2.2 Health impact assessment using deep learning

Deep learning has emerged as a powerful tool for assessing the health impacts of environmental exposures (27). By leveraging large-scale health datasets and advanced neural network architectures, researchers have made significant progress in understanding the complex interactions between the exposome and human health. These models have been applied to a wide range of health outcomes, including respiratory diseases, cardiovascular conditions, and mental health disorders. One of the primary applications of deep learning in health impact assessment is in the prediction of disease risk based on environmental exposure data (28). For example, neural networks have been used to model the relationship between air pollution and respiratory diseases, leveraging spatial and temporal data on pollutant concentrations. CNNs have been particularly effective in capturing spatial patterns of exposure, while LSTMs have been used to account for temporal trends in health outcomes. These models often outperform traditional statistical approaches, such as generalized linear models, by capturing non-linear and complex interactions between variables (29). Deep learning models have been used for causal inference in health impact studies (30). Techniques such as deep reinforcement learning and generative adversarial networks (GANs) have been explored for simulating counterfactual scenarios, enabling researchers to estimate the causal effects of environmental interventions (31). For instance, GANs have been employed to generate synthetic data for underrepresented populations, improving the generalizability of health impact assessments. Attention mechanisms and explainability frameworks have been integrated into these models to enhance their interpretability, facilitating their application in policy-making and public health interventions (32). Another important area of research is the integration of genetic and epigenetic data into deep learning models for exposome-health studies (33). Multi-modal deep learning approaches have been developed to combine environmental, genetic, and clinical data, providing a comprehensive understanding of gene-environment interactions. These models have been used to investigate how environmental exposures influence epigenetic modifications and, in turn, contribute to disease risk. For example, deep autoencoders have been applied to identify patterns in DNA methylation data associated with air pollution exposure, shedding light on potential biological pathways underlying health effects (34). Challenges persist in the application of deep learning to health impact assessment. One major limitation is the availability of high-quality, large-scale datasets that integrate environmental exposures and health outcomes. Data privacy concerns and ethical considerations further complicate data sharing and integration (35). Another challenge is the need for robust validation frameworks to ensure the reliability and reproducibility of deep learning models in diverse populations and settings. Addressing these challenges will require collaborative efforts across disciplines, including environmental science, epidemiology, and computer science.

### 2.3 Spatiotemporal modeling for environmental health

Spatiotemporal modeling has emerged as a key area of interest in applying deep learning techniques to environmental health studies (36). The dynamic and spatially heterogeneous nature of environmental exposures necessitates the use of models that can capture both spatial and temporal variations in data. Deep learning models, such as spatiotemporal convolutional networks and graph neural networks (GNNs), have shown great potential in this regard, enabling researchers to analyze complex patterns in environmental and health datasets. Spatiotemporal convolutional networks, which combine convolutional operations with temporal processing, have been widely used for exposure prediction and monitoring (37). These models can capture fine-grained spatial patterns while accounting for temporal dependencies in data. For instance, spatiotemporal deep learning has been employed to model urban air quality, combining inputs such as traffic data, meteorological variables, and satellite imagery. These models are capable of predicting pollutant concentrations at high spatial resolutions and short time intervals, providing critical insights for environmental health studies. Graph neural networks (GNNs) have also gained attention for their ability to model relational data and spatial dependencies in environmental health research (38). GNNs represent data as graphs, with nodes corresponding to spatial locations and edges capturing spatial or functional relationships. This approach has been used to model disease transmission networks, assess the spread of infectious diseases, and evaluate the spatial distribution of environmental exposures. GNNs are particularly useful for integrating data from diverse sources, such as combining satellite-derived air quality estimates with social determinants of health. Another area of advancement is the use of attention mechanisms in spatiotemporal modeling (39). Attention mechanisms allow models to concentrate on the most pertinent features or areas within the data, enhancing both their interpretability and effectiveness. For example, attention-based spatiotemporal models have been applied to identify hotspots of environmental exposures and their associated health impacts. These models can provide actionable insights for public health interventions by highlighting areas with the greatest need for environmental mitigation efforts. Despite these advancements, spatiotemporal modeling in environmental health faces several challenges (40). One key issue is the computational complexity of processing high-resolution spatiotemporal data, which often requires significant computational resources and expertise in parallel processing. Another challenge is the scalability of these models to large geographic areas and diverse populations, as well as the need for robust methods to handle missing or noisy data. Future research efforts should focus on developing efficient algorithms and scalable architectures to address these limitations. Interdisciplinary collaborations will be essential for advancing the application of spatiotemporal deep learning models in environmental health research.

## 3 Method

### 3.1 Overview

The exposome represents the comprehensive set of environmental exposures an individual encounters throughout their lifetime, encompassing chemical, physical, and social factors. High-resolution exposome research aims to systematically capture and analyze these exposures at an unprecedented spatial and temporal granularity, bridging the gap between environment-wide exposure assessments and individual-level health outcomes. This paper introduces a novel methodological framework to enhance the precision and scalability of exposome quantification through the integration of advanced computational models, multi-modal data fusion, and adaptive optimization strategies. Our approach is organized into three key components: In Section 3.2, we present the foundational preliminaries required to formalize the problem of high-resolution exposome modeling. This involves defining the mathematical representation of exposures, their spatiotemporal characteristics, and the associated uncertainties. In Section 3.3, we describe a newly developed model designed to effectively capture the complex, multi-scale dynamics of environmental exposures. This model incorporates hierarchical structures and integrates diverse datasets, such as remote sensing imagery, personal monitoring data, and geospatial analytics. In Section 3.6, we propose an innovative strategy that leverages domain-specific knowledge and optimization principles to adaptively refine the exposure quantification process. This strategy enhances computational efficiency while maintaining high accuracy across varying exposure scenarios.

Central to our framework is the seamless fusion of data streams from heterogeneous sources, ranging from high-resolution satellite imagery to wearable sensors. This integration is achieved through a robust pipeline that standardizes, harmonizes, and preprocesses multi-modal data, ensuring consistency and compatibility for downstream analysis. Our approach incorporates novel statistical techniques to account for uncertainties and missing data, thus enabling more reliable inferences about exposure profiles. The overarching goal of our method is to bridge the traditional limitations of exposome research by introducing a scalable, adaptive, and high-resolution framework. Through this work, we aim to empower researchers and policymakers with actionable insights into the intricate interactions between environmental factors and health outcomes, ultimately advancing the understanding of environmental determinants of health.

### 3.2 Preliminaries

High-resolution exposome modeling involves capturing the dynamic and multi-dimensional nature of environmental exposures across both spatial and temporal dimensions. To formalize this problem, we begin by introducing the key notations, definitions, and mathematical constructs that will serve as the foundation for the proposed framework.

Let the exposome be represented as a set $E=\left\{e_{1},e_{2},\ldots ,e_{N}\right\}$, where each *e*_*i*_ denotes a specific environmental exposure. Each exposure *e*_*i*_ is modeled as a time-dependent and spatially distributed variable defined over a domain $D\subseteq {\mathbb{R}}^{2}\times \text{𝕋}$, where ℝ^2^ represents the geographical space and 𝕋 represents time. Thus, *e*_*i*_ can be expressed as:


(1) $$\begin{array}{l} e_{i}\left(x,t\right):D\to \mathbb{R}, \end{array}$$

where *x* ∈ ℝ^2^ is the spatial location, *t* ∈ 𝕋 is the time index, and *e*_*i*_(*x, t*) provides the exposure intensity of *e*_*i*_ at location *x* and time *t*.

Environmental exposures inherently exhibit variability across multiple spatial and temporal scales. To capture this, we introduce a multi-scale decomposition for *e*_*i*_(*x, t*):


(2) $$\begin{array}{l} e_{i}\left(x,t\right)=\overset{L}{\underset{l=1}{\sum }}\overset{H}{\underset{h=1}{\sum }}e_{i}^{\left(l,h\right)}\left(x,t\right), \end{array}$$

where $e_{i}^{\left(l,h\right)}\left(x,t\right)$ represents the contribution of exposure *e*_*i*_ at spatial scale *l* and temporal scale *h*. Here, *L* denotes the number of spatial scales, and *H* denotes the number of temporal resolutions.

The observed data for exposures often come from multiple sources, such as satellite remote sensing, wearable devices, and fixed monitoring stations. Let O denote the set of observed data streams, where each $O_{k}\in O$ corresponds to a particular data modality. The observed exposure $e_{i}^{\text{obs}}$ is then related to the true exposure *e*_*i*_ through a measurement model:


(3) $$\begin{array}{l} e_{i}^{\text{obs}}\left(x,t\right)=H_{k}\left(e_{i}\left(x,t\right)\right)+\eta_{k}\left(x,t\right), \end{array}$$

where $H_{k}$ is the observation operator for the *k*-th data modality, and η_*k*_(*x, t*) represents the noise associated with the measurement process. Different observation operators capture the resolution and biases unique to the modality.

Given the heterogeneity of data sources, it is essential to account for uncertainties in the measurements. Let U represent the uncertainty associated with the exposome. For a given exposure *e*_*i*_, we define its uncertainty as a probability distribution $\mathbb{P}\left(e_{i}|O\right)$, which can be derived using Bayesian principles:


(4) $$\begin{array}{l} \mathbb{P}\left(e_{i}\left(x,t\right)|O\right)\propto \mathbb{P}\left(O|e_{i}\left(x,t\right)\right)\cdot \mathbb{P}\left(e_{i}\left(x,t\right)\right), \end{array}$$

where $\mathbb{P}\left(O|e_{i}\left(x,t\right)\right)$ is the likelihood of the observations given the true exposure, and ℙ(*e*_*i*_(*x, t*)) represents the prior knowledge about the exposure distribution.

The task of high-resolution exposome modeling is to infer *e*_*i*_(*x, t*) from the observed data O by solving an optimization problem that minimizes the reconstruction error while accounting for uncertainties:


(5) e^i(x,t)=arg minei[∑k∈O‖Hk(ei(x,t))−eiobs(x,t)‖22+λR(ei(x,t))],

where $R\left(e_{i}\left(x,t\right)\right)$ is a regularization term that incorporates spatial or temporal smoothness constraints, and λ is a hyperparameter controlling the regularization strength.

### 3.3 Adaptive multi-scale exposome network (AMSEN)

**Figure 1:**
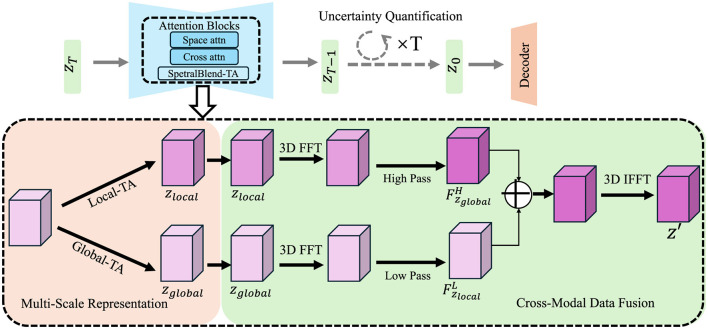
Diagram illustrating the Adaptive Multi-Scale Exposome Network (AMSEN) framework. The figure highlights the multi-scale representation using Local and Global Temporal Attention (Local-TA and Global-TA), 3D Fourier Transforms (FFT), and cross-modal data fusion for high-resolution exposure modeling. The upper section emphasizes uncertainty quantification with attention blocks, including space, cross, and spectral attention mechanisms, enabling robust predictions with confidence-aware outputs. The integration of hierarchical features and inverse FFT (IFFT) ensures accurate reconstruction across spatial and temporal scales.

To address the challenges inherent in high-resolution exposome modeling, we propose the Adaptive Multi-Scale Exposome Network (AMSEN), a novel computational framework designed to capture the complex, multi-scale dynamics of environmental exposures. AMSEN is built on a hierarchical architecture that integrates spatial, temporal, and cross-modal information from diverse data sources to achieve robust exposure quantification (As shown in Figure 1).

#### 3.3.1 Multi-scale representation

Environmental exposures exhibit variability at multiple spatial and temporal scales. AMSEN incorporates a hierarchical representation to model this variability and captures the complex dynamics of exposure interactions. The spatial domain ℝ^2^ is discretized into a set of *L* nested grids ${\left\{G_{l}\right\}}_{l=1}^{L}$, where each $G_{l}$ represents a resolution level. Each grid $G_{l}$ partitions the spatial domain into cells of size Δ*x*_*l*_ × Δ*y*_*l*_, where the resolution becomes finer with increasing *l*. This hierarchical spatial discretization enables the model to encode both coarse-grained and fine-grained spatial structures.

The temporal domain *T* is discretized into a set of *H* nested intervals ${\left\{T_{h}\right\}}_{h=1}^{H}$, corresponding to different temporal resolutions. Each temporal interval $T_{h}$ is defined by its duration Δ*t*_*h*_, where finer temporal resolutions correspond to smaller Δ*t*_*h*_. This nested structure enables AMSEN to adapt to both short-term variations and long-term trends in environmental exposures.

For each exposure *e*_*i*_, AMSEN constructs a multi-scale representation $e_{i}^{\left(l,h\right)}\left(x,t\right)$, which captures the exposure intensity at spatial scale *l* and temporal scale *h*. The overall exposure representation is defined as a sum over all scales:


(6) $$\begin{array}{l} e_{i}\left(x,t\right)=\overset{L}{\underset{l=1}{\sum }}\overset{H}{\underset{h=1}{\sum }}e_{i}^{\left(l,h\right)}\left(x,t\right), \end{array}$$

where $e_{i}^{\left(l,h\right)}\left(x,t\right)$ represents the contribution of exposure *e*_*i*_ at spatial resolution *l* and temporal resolution *h*. To construct $e_{i}^{\left(l,h\right)}\left(x,t\right)$, the model applies a series of operations over spatial and temporal neighborhoods.

The spatial contribution is computed using convolutional operations on the grid $G_{l}$, where the receptive field increases with *l*. Let *K*_*l*_ represent the convolutional kernel for scale *l*, and let *N*_*l*_(*x*) denote the spatial neighborhood of location *x* on grid $G_{l}$. Then, the spatial feature extraction is given by:


(7) $$\begin{array}{l} e_{i}^{\left(l\right)}\left(x,t\right)=\underset{x^{\prime }\in N_{l}\left(x\right)}{\sum }K_{l}\left(x,x^{\prime }\right)\cdot e_{i}\left(x^{\prime },t\right), \end{array}$$

where $K_{l}\left(x,x^{\prime }\right)$ encodes the spatial relationship between *x* and *x*′ at scale *l*. This operation aggregates information from the spatial neighborhood and encodes it into the multi-scale representation.

For the temporal contribution, a similar approach is applied using temporal kernels *K*_*h*_ over the temporal intervals $T_{h}$. Let *N*_*h*_(*t*) denote the temporal neighborhood of time *t* within interval $T_{h}$. The temporal feature extraction is given by:


(8) $$\begin{array}{l} e_{i}^{\left(h\right)}\left(x,t\right)=\underset{t^{\prime }\in N_{h}\left(t\right)}{\sum }K_{h}\left(t,t^{\prime }\right)\cdot e_{i}\left(x,t^{\prime }\right). \end{array}$$

### 3.4 Cross-modal data fusion

AMSEN integrates diverse data sources, including satellite imagery, wearable sensors, and monitoring stations, by constructing modality-specific feature extraction pathways. Let $O_{k}$ denote the observation from modality *k*. AMSEN uses a shared encoder-decoder architecture to extract features Φ_*k*_(*x, t*) from each modality:


(9) $$\begin{array}{l} {\text{Φ}}_{k}\left(x,t\right)=F_{k}\left(e_{i}^{\text{obs}}\left(x,t\right);\theta_{k}\right), \end{array}$$

where $F_{k}$ is the modality-specific feature extractor parameterized by θ_*k*_, and $e_{i}^{\text{obs}}\left(x,t\right)$ represents the input signal for modality *k* at spatial location *x* and temporal instance *t*. These extracted features Φ_*k*_(*x, t*) encode modality-specific information while preserving the unique characteristics of the corresponding data source.

The features extracted from all modalities are subsequently fused in a shared latent space Z. The fusion step combines information across multiple modalities to achieve a comprehensive representation:


(10) $$Z(x,t)=\text{Fuse}({\text{Φ}}_{1}(x,t),{\text{Φ}}_{2}(x,t),\ldots ,{\text{Φ}}_{K}(x,t)),$$

where *K* represents the number of modalities, and Fuse(·) is the fusion operator. This operator can take various forms, such as concatenation:


(11) $$\begin{array}{l} \text{Fuse}\left({\text{Φ}}_{1},{\text{Φ}}_{2},\ldots ,{\text{Φ}}_{K}\right)=\text{Concat}\left({\text{Φ}}_{1},{\text{Φ}}_{2},\ldots ,{\text{Φ}}_{K}\right), \end{array}$$

or attention mechanisms for weighted feature integration:


(12) $$\begin{array}{l} \text{Fuse}\left({\text{Φ}}_{1},{\text{Φ}}_{2},\ldots ,{\text{Φ}}_{K}\right)=\overset{K}{\underset{k=1}{\sum }}\alpha_{k}\cdot {\text{Φ}}_{k}, \end{array}$$

where α_*k*_ are the attention weights learned to prioritize the contribution of each modality.

The fused latent representation Z(x,t) serves as the input for reconstructing the high-resolution exposure field. The reconstruction is performed by a decoder G parameterized by ψ, which maps Z to the predicted exposure field:


(13) $$\begin{array}{l} {ê}_{i}\left(x,t\right)=G\left(Z\left(x,t\right);\psi \right). \end{array}$$

The decoder G employs a multi-layer architecture to refine and upscale the fused latent representation Z(x,t), ensuring accurate reconstruction of spatial and temporal exposure patterns.

### 3.5 Uncertainty quantification

**Figure 2:**
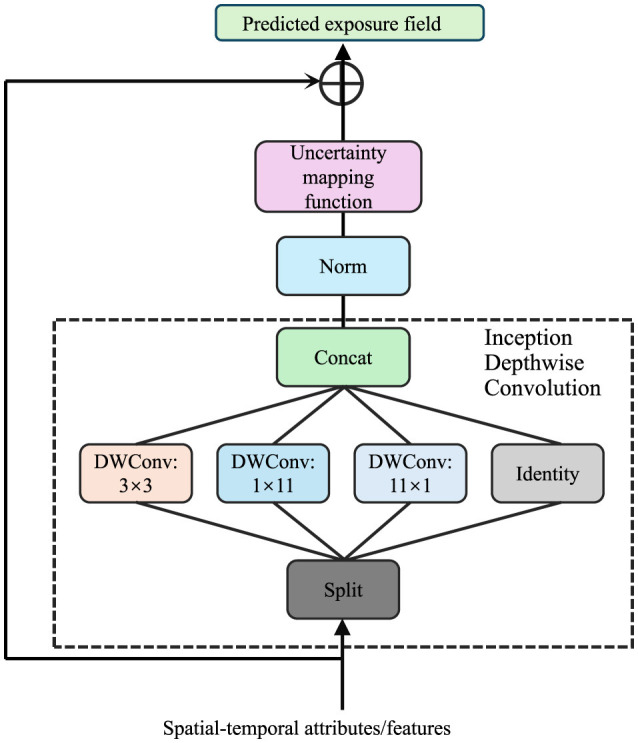
Diagram of uncertainty quantification framework, illustrating the integration of an uncertainty mapping function, normalization, and Inception Depthwise Convolution modules to process spatial-temporal attributes and predict exposure fields with associated uncertainty estimates.

AMSEN explicitly quantifies uncertainty in exposure estimates by introducing a probabilistic modeling layer. Let ê_*i*_(*x, t*) represent the predicted exposure field, which is subject to inherent variability and uncertainty due to factors such as sensor noise, data sparsity, and environmental dynamics (As shown in Figure 2).

To characterize the uncertainty associated with the predictions, AMSEN models the variance $\sigma_{i}^{2}\left(x,t\right)$ as a function of the spatial-temporal context, described by:


(14) $$\begin{array}{l} \sigma_{i}^{2}\left(x,t\right)=\text{Var}\left[{ê}_{i}\left(x,t\right)\right]=H\left(Z\left(x,t\right);\phi \right), \end{array}$$

where H is a learned uncertainty mapping function parameterized by ϕ. The input to H is Z(x,t), a feature representation encoding the spatial-temporal attributes of the environment, which could include meteorological data, geographic features, and sensor characteristics.

To ensure that the uncertainty estimates are robust and meaningful, AMSEN employs a probabilistic framework during model training. The predictive distribution of ê_*i*_(*x, t*) is assumed to follow a Gaussian distribution:


(15) $$\begin{array}{l} {ê}_{i}\left(x,t\right)\sim N\left(\mu_{i}\left(x,t\right),\sigma_{i}^{2}\left(x,t\right)\right), \end{array}$$

where μ_*i*_(*x, t*) is the predicted mean exposure field and $\sigma_{i}^{2}\left(x,t\right)$ is the predicted variance representing the uncertainty. The model is trained by maximizing the likelihood of the observed data under this probabilistic model, which is equivalent to minimizing the negative log-likelihood (NLL) loss:


(16) $$\begin{array}{l} L_{\text{NLL}}=\frac{1}{N}\overset{N}{\underset{i=1}{\sum }}\left[\frac{{\left(y_{i}-\mu_{i}\left(x,t\right)\right)}^{2}}{2\sigma_{i}^{2}\left(x,t\right)}+\frac{1}{2}\log\sigma_{i}^{2}\left(x,t\right)\right], \end{array}$$

where *y*_*i*_ represents the ground truth exposure measurements, and *N* is the total number of observations. This loss function encourages the model to accurately predict both the mean and variance of the exposure field while penalizing overconfident or underconfident uncertainty estimates.

In addition, AMSEN incorporates regularization mechanisms to ensure stable training of the uncertainty mapping. For instance, a prior constraint can be imposed on σ_*i*_(*x, t*) to prevent pathological cases where the uncertainty collapses to zero or diverges to infinity. A commonly used regularization term is:


(17) $$\begin{array}{l} L_{\text{reg}}=\text{λ}\cdot \text{𝔼}\left[\sigma_{i}^{2}\left(x,t\right)\right], \end{array}$$

where λ is a hyperparameter controlling the strength of the regularization.

### 3.6 Adaptive exposure optimization strategy (AEOS)

**Figure 3:**
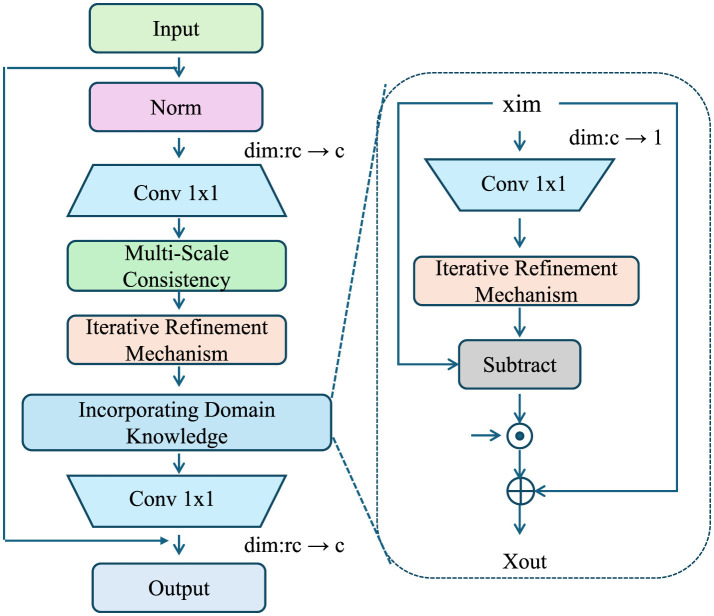
Illustration of the Adaptive Exposure Optimization Strategy (AEOS) framework, showcasing the multi-scale consistency, iterative refinement mechanism, and domain knowledge integration for robust exposure modeling. The left part depicts the main pipeline with input normalization, convolutional layers, domain knowledge incorporation, and iterative refinements. The right inset details the iterative refinement mechanism, emphasizing residual updates and refinement feedback.

To complement the proposed Adaptive Multi-Scale Exposome Network (AMSEN), we introduce the Adaptive Exposure Optimization Strategy (AEOS), a novel approach designed to dynamically refine exposure estimation by leveraging domain-specific knowledge, spatiotemporal regularities, and adaptive computational principles. AEOS enhances the robustness, interpretability, and scalability of AMSEN, ensuring its applicability across diverse real-world scenarios (As shown in Figure 3).

#### 3.6.1 Incorporating domain knowledge

Environmental exposure modeling often benefits significantly from integrating domain-specific knowledge, such as regulatory limits, spatial boundaries, pollutant dispersion dynamics, and chemical transformation rates. This knowledge provides meaningful priors that guide the model toward physically plausible and scientifically consistent solutions. In AEOS, these priors are incorporated as soft constraints during the optimization process to balance empirical observations with domain-based expectations.

For each exposure *e*_*i*_, we define a prior distribution ℙ(*e*_*i*_) based on domain knowledge. The prior distribution imposes a probabilistic structure over the exposure field, which can be represented as:


(18) $$\begin{array}{l} \mathbb{P}\left(e_{i}\left(x,t\right)\right)\propto \exp-C\left(e_{i}\left(x,t\right)\right), \end{array}$$

where $C\left(e_{i}\left(x,t\right)\right)$ is a penalty function that quantifies deviations of *e*_*i*_(*x, t*) from known environmental constraints. Examples of such constraints include maximum allowable pollutant concentrations, pollutant dispersion patterns governed by physical laws, and decay rates under chemical or biological processes. For instance, $C\left(e_{i}\left(x,t\right)\right)$ may include terms such as:


(19) $$\begin{array}{l} C\left(e_{i}\left(x,t\right)\right)=\alpha \cdot \max{\left(0,e_{i}\left(x,t\right)-e_{\text{max}}\right)}^{2}+\beta \cdot ||\nabla e_{i}\left(x,t\right)-\text{v}\left(x,t\right)\cdot \nabla c{||}^{2}, \end{array}$$

where *e*_max_ is a regulatory limit for exposure, **v**(*x, t*) represents the local wind or flow vector field, and ∇*c* denotes the expected concentration gradient due to advection-diffusion dynamics. The parameters α and β control the relative weight of different penalty components.

These prior constraints are incorporated into AMSEN's optimization framework by introducing a regularization term in the loss function. The regularization term penalizes the model for deviating from the prior distribution:


(20) $$\begin{array}{l} L_{\text{prior}}={\text{λ}}_{\text{prior}}\int C\left(e_{i}\left(x,t\right)\right)dxdt, \end{array}$$

where λ_prior_ is a hyperparameter that determines the strength of the prior enforcement. A higher value of λ_prior_ results in stricter adherence to the domain knowledge, while a lower value allows the model to prioritize empirical data.

To ensure flexibility and adaptability, AMSEN uses learnable parameters within the prior penalty functions. For example, instead of fixing **v**(*x, t*), the model can learn an estimated flow field $\hat{\text{v}}\left(x,t\right)$ that is consistent with observed exposure patterns. The penalty function then becomes:


(21) $$\begin{array}{l} C\left(e_{i}\left(x,t\right)\right)=\alpha \cdot \max{\left(0,e_{i}\left(x,t\right)-e_{\text{max}}\right)}^{2}+\beta \cdot ||\nabla e_{i}\left(x,t\right)-\hat{\text{v}}\left(x,t\right)\cdot \nabla c{||}^{2}. \end{array}$$

Another important source of domain knowledge is pollutant decay over time. For many pollutants, the decay process can be approximated using first-order kinetics, represented as:


(22) $$\begin{array}{l} \frac{\partial e_{i}\left(x,t\right)}{\partial t}=-k\cdot e_{i}\left(x,t\right), \end{array}$$

where *k* is the decay rate constant. This physical law can be directly encoded into the prior penalty function by enforcing consistency with the temporal decay dynamics:


(23) $$C_{\text{decay}}(e_{i}(x,t))=\gamma \cdot {\left\|\frac{\partial e_{i}(x,t)}{\partial t}+k\cdot e_{i}(x,t)\right\|}^{2},$$

where γ controls the strength of the decay consistency term.

#### 3.6.2 Iterative refinement mechanism

AEOS incorporates an iterative refinement mechanism that adapts to the spatiotemporal heterogeneity of exposure patterns. Let R(x,t) represent a residual field that captures unresolved variability in exposure estimates:


(24) $$\begin{array}{l} R\left(x,t\right)=e_{i}^{\text{obs}}\left(x,t\right)-{ê}_{i}\left(x,t\right), \end{array}$$

where $e_{i}^{\text{obs}}\left(x,t\right)$ is the observed exposure value, and ê_*i*_(*x, t*) is the model-predicted estimate at spatial location *x* and temporal instance *t*. The residual R(x,t) quantifies the discrepancy between the observed and predicted values, serving as the basis for iterative updates.

At each iteration *k*, AEOS updates the exposure estimate ${ê}_{i}^{\left(k\right)}\left(x,t\right)$ to reduce the residual field. The update rule is defined as:


(25) $$\begin{array}{l} {ê}_{i}^{\left(k+1\right)}\left(x,t\right)={ê}_{i}^{\left(k\right)}\left(x,t\right)+\alpha R^{\left(k\right)}\left(x,t\right), \end{array}$$

where α is a learning rate that controls the step size of refinement, and $R^{\left(k\right)}\left(x,t\right)$ is the residual field computed at iteration *k*. The iterative process continues until the norm of the residual, $||R^{\left(k\right)}\left(x,t\right)|{|}_{2}$, falls below a predefined convergence threshold ϵ:


(26) $$\begin{array}{l} ||R^{\left(k\right)}\left(x,t\right){||}_{2}\leq \epsilon . \end{array}$$

AEOS incorporates an adaptive convergence criterion that varies spatially and temporally based on the complexity of exposure patterns. For regions with high variability, a stricter convergence threshold is applied to ensure accuracy:


(27) $$\begin{array}{l} \epsilon_{j}=\epsilon_{0}+\beta ||\nabla e_{i}\left(x,t\right){||}_{2}, \end{array}$$

where ϵ_0_ is the baseline threshold, β is a scaling factor, and ||∇*e*_*i*_(*x, t*)||_2_ represents the magnitude of the exposure gradient, which indicates local variability.

To improve computational efficiency, AEOS dynamically allocates computational resources based on exposure variability and data density. The spatial domain $D\subseteq {\mathbb{R}}^{2}$ is partitioned into regions ${\left\{D_{j}\right\}}_{j=1}^{M}$ based on exposure gradients:


(28) $$\begin{array}{l} D_{j}=\left\{x\in D|||\nabla e_{i}\left(x,t\right){||}_{2}\in \left[g_{j},g_{j+1}\right)\right\}, \end{array}$$

where *g*_*j*_ represents the gradient threshold for region *j*, and *M* is the total number of regions. High-gradient regions, which exhibit rapid spatial changes in exposure, are assigned more computational resources to capture these local variations effectively.

The computational resource allocation for each region $D_{j}$ is given by:


(29) $$\begin{array}{l} C_{j}\propto ||\nabla e_{i}\left(x,t\right){||}_{2}+\rho d_{j}, \end{array}$$

where *C*_*j*_ represents the computational budget assigned to region $D_{j}$, ρ is a balancing factor, and *d*_*j*_ denotes the data density within $D_{j}$. Regions with higher exposure gradients ||∇*e*_*i*_(*x, t*)||_2_ or higher data density *d*_*j*_ are prioritized to ensure accurate and efficient modeling.

The iterative refinement mechanism is further enhanced through a multi-scale approach. Exposure estimates are refined across a hierarchy of spatial resolutions. Let ℓ ∈ {1, 2, …, *L*} denote the resolution level, where finer levels capture detailed local patterns, and coarser levels capture broader trends. The refinement at each resolution is expressed as:


(30) $$\begin{array}{l} {ê}_{i}^{\left(\ell +1\right)}\left(x,t\right)={ê}_{i}^{\left(\ell \right)}\left(x,t\right)+\alpha^{\left(\ell \right)}R^{\left(\ell \right)}\left(x,t\right), \end{array}$$

where α^(ℓ)^ is the learning rate specific to resolution level ℓ, and $R^{\left(\ell \right)}\left(x,t\right)$ is the residual field computed at that resolution.

#### 3.6.3 Multi-scale consistency

**Figure 4:**
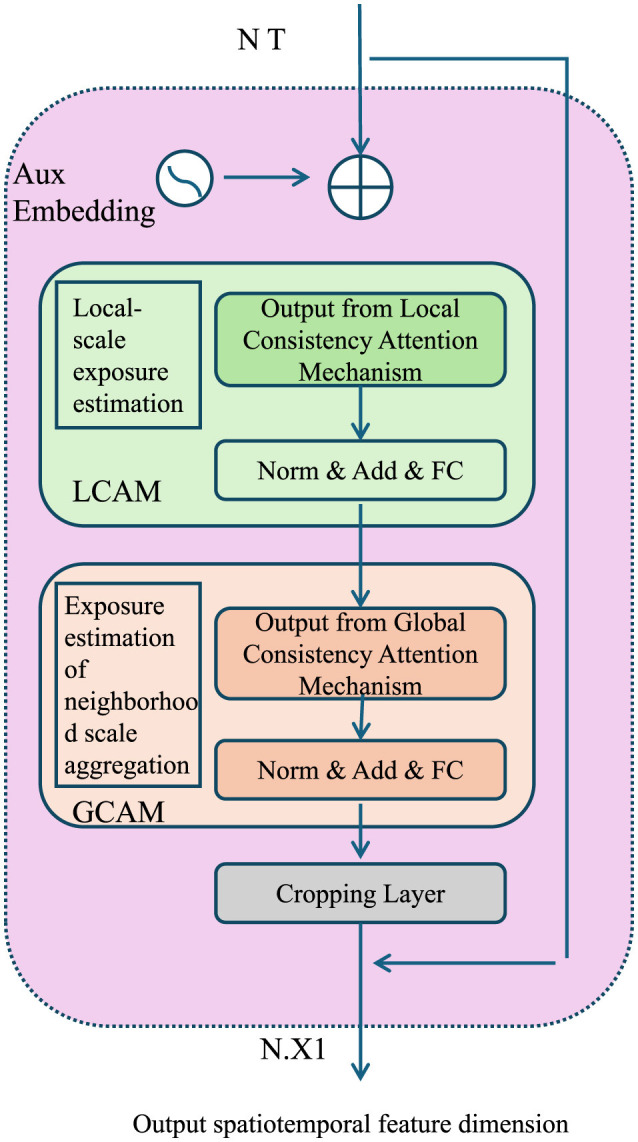
Diagram showcasing the Multi-Scale Consistency framework, including the Local Consistency Attention Mechanism (LCAM) and Global Consistency Attention Mechanism (GCAM). The figure illustrates the integration of local-scale exposure estimation and global neighborhood aggregation, leveraging auxiliary embeddings, attention mechanisms, and normalization layers to ensure coherence across spatial and temporal scales. Outputs are harmonized and refined to optimize spatiotemporal feature dimensions.

AEOS enforces consistency across spatial and temporal scales by introducing a scale-harmonization term in the optimization process (As shown in Figure 4).

Let $e_{i}^{\left(l,h\right)}\left(x,t\right)$ denote the exposure estimate at spatial scale *l* and temporal scale *h*. The consistency term is defined as:


(31) $$L_{\text{scale}}=\sum_{l=1}^{L}\sum_{h=1}^{H}{\left\|e_{i}^{\left(l,h\right)}(x,t)-{\bar{e}}_{i}^{\left(l,h\right)}(x,t)\right\|}_{2}^{2},$$

where ${ē}_{i}^{\left(l,h\right)}\left(x,t\right)$ is the aggregated estimate across neighboring scales. This aggregation is performed through a weighted averaging mechanism, incorporating both spatial and temporal dependencies. ${ē}_{i}^{\left(l,h\right)}\left(x,t\right)$ can be computed as:


(32) $$\begin{array}{l} {ē}_{i}^{\left(l,h\right)}\left(x,t\right)=\frac{\sum_{\left(l^{\prime },h^{\prime }\right)\in N\left(l,h\right)}w_{l^{\prime },h^{\prime }}e_{i}^{\left(l^{\prime },h^{\prime }\right)}\left(x,t\right)}{\sum_{\left(l^{\prime },h^{\prime }\right)\in N\left(l,h\right)}w_{l^{\prime },h^{\prime }}}, \end{array}$$

where N(l,h) denotes the set of neighboring scales, and $w_{l^{\prime },h^{\prime }}$ are scale-dependent weights that reflect the relative importance of each neighboring scale. These weights are learned during training to adaptively balance contributions from coarse- and fine-scale information. By minimizing $L_{\text{scale}}$, the model ensures coherence across scales, reducing artifacts and inconsistencies introduced by transitions between resolutions.

To further enhance performance, AEOS leverages the uncertainty estimates produced by AMSEN to guide decision-making and prioritize refinement efforts. Let σ_*i*_(*x, t*) denote the predicted uncertainty for exposure *e*_*i*_. AEOS identifies high-uncertainty regions as:


(33) $$\begin{array}{l} U=\left\{\left(x,t\right)|\sigma_{i}\left(x,t\right)>\tau \right\}, \end{array}$$

where τ is an uncertainty threshold, typically chosen based on a desired confidence level or domain-specific requirements. Regions in U are prioritized for additional refinement by reallocating computational resources or employing higher-resolution models to reduce uncertainty. This process is formalized as a resource-constrained optimization problem, where the goal is to minimize the overall uncertainty within a fixed computational budget *B*:


(34) $$\begin{array}{l} \underset{R}{\min}\underset{\left(x,t\right)\in R}{\sum }\sigma_{i}\left(x,t\right),\;\text{subject to}\;\underset{\left(x,t\right)\in R}{\sum }c\left(x,t\right)\leq B, \end{array}$$

where R⊆U is the set of refined regions, and *c*(*x, t*) is the computational cost associated with refining (*x, t*). Dynamic programming techniques are used to solve this optimization problem efficiently, enabling the model to allocate resources where they are most needed.

The complete optimization problem solved by AEOS is formulated as a composite objective that balances reconstruction accuracy, prior enforcement, multi-scale consistency, and computational efficiency:


(35) $$\begin{array}{l} L_{\text{AEOS}}=L_{\text{recon}}+{\text{λ}}_{\text{prior}}L_{\text{prior}}+{\text{λ}}_{\text{scale}}L_{\text{scale}}+{\text{λ}}_{\text{uncertainty}}L_{\text{uncertainty}}, \end{array}$$

where $L_{\text{recon}}$ is the reconstruction loss, $L_{\text{prior}}$ enforces domain-specific constraints or regularizations, and $L_{\text{uncertainty}}$ represents the penalty for high uncertainty regions that are left unrefined. Each term is weighted by its corresponding hyperparameter λ_prior_, λ_scale_, and λ_uncertainty_ to control its relative contribution during training.

In our framework, AMSEN and AEOS are designed as complementary components that work in tandem to address the challenges of modeling complex, high-dimensional exposome data and their associated health risks. AMSEN serves as the representation backbone, constructing hierarchical spatial-temporal embeddings of environmental exposures. It leverages multi-scale convolutional modules and spectral attention to capture both localized and global patterns in exposure distributions. These embeddings encode structural heterogeneity and latent dependencies across space and time, which are critical for accurate exposure assessment. On top of AMSEN, AEOS (Adaptive Exposure Optimization Strategy) acts as a decision-level module that refines and calibrates exposure-driven predictions. Specifically, AEOS takes the intermediate outputs from AMSEN and applies uncertainty-aware selection mechanisms to identify regions or instances where the model's confidence is low or the input signal is noisy. Based on these uncertainty estimates, AEOS adaptively adjusts the contribution of each spatial or temporal component during downstream health outcome prediction, effectively re-weighting and optimizing the learned representations. This layered design allows AMSEN to focus on extracting informative and expressive features, while AEOS enhances reliability and robustness through guided optimization. Together, they form an integrated pipeline where exposure modeling and outcome prediction are tightly coupled, ensuring the model can generalize across varying data quality levels and population contexts.

## 4 Experimental setup

### 4.1 Dataset

The ProcGen Dataset (41) is a procedurally generated benchmark suite designed for evaluating the generalization capabilities of reinforcement learning algorithms. It offers a diverse set of tasks that are automatically generated, ensuring variability across different runs and preventing overfitting to specific levels or instances. The dataset contains environments that test various aspects of agent learning, such as exploration, memory, and planning, making it a valuable resource for studying generalization in complex, dynamic scenarios. The GBD Dataset (42) is a large-scale benchmark designed for graph-based deep learning tasks. It provides graph structures with varying node and edge attributes to evaluate the performance of algorithms on tasks such as node classification, graph classification, and link prediction. The dataset spans multiple domains, including social networks, biological networks, and knowledge graphs, providing a comprehensive evaluation framework for graph neural networks. The OpenStreetMap Dataset (43) is a rich geospatial dataset derived from the OpenStreetMap project. It consists of detailed geographic information, including road networks, building footprints, and point-of-interest data. The dataset is widely used for tasks such as map generation, route optimization, and urban planning. Its open and collaborative nature ensures a broad and up-to-date coverage of global geographic data, making it a vital resource for geospatial research and applications. The SUMO Dataset (44) is a traffic simulation dataset based on the Simulation of Urban Mobility (SUMO) platform. It includes synthetic and real-world traffic scenarios, enabling the evaluation of traffic modeling, control, and prediction algorithms. The dataset supports high-fidelity simulations with diverse configurations of road networks, traffic signals, and vehicle behaviors, making it essential for studying intelligent transportation systems and autonomous driving technologies.

To emulate realistic exposome scenarios within the context of reinforcement learning datasets, we simulated environmental exposure variables by extracting and transforming domain-relevant spatial and behavioral features. In the ProcGen Dataset, procedurally generated terrain features, obstacle distributions, and agent-environment interactions were encoded as analogs of environmental exposures such as noise level, visual complexity, and physical obstruction density. In the SUMO traffic dataset, traffic flow rates, vehicle density, and route proximity were mapped to exposure proxies representing air pollution intensity and noise gradients. For the GBD and OpenStreetMap Datasets, we utilized node-level geospatial features such as land-use type, population density, and connectivity as inputs for constructing exposure fields akin to urban design, socio-environmental stress, and green space accessibility. All features were preprocessed through normalization, spatial resampling, and temporal slicing to produce multi-resolution tensors aligned with our hierarchical model design. To mimic real-world uncertainties in sensor-based exposome data, we introduced stochastic perturbations using Gaussian noise and synthetic temporal drift in select modalities. Additionally, a unified spatiotemporal grid system was applied to synchronize data from diverse sources, enabling effective fusion across modalities in the AMSEN framework. This simulation protocol ensures that although the datasets originate from artificial or semi-synthetic environments, they structurally resemble the challenges faced in real-world exposome modeling—namely heterogeneity, sparsity, and spatiotemporal misalignment.

### 4.2 Experimental details

Our experiments are designed to evaluate the performance and robustness of the proposed method across diverse datasets and application scenarios. All models are implemented using PyTorch and trained on NVIDIA A100 GPUs. The batch size is set to 128 for all datasets, and the Adam optimizer is employed with an initial learning rate of 0.001. The learning rate is adjusted dynamically using a cosine annealing schedule, which decays over the course of training. For regularization, dropout with a probability of 0.3 is applied to the fully connected layers to mitigate overfitting. Weight decay is set to 10^−5^ to further control overfitting and improve generalization. Training is performed for 200 epochs on all datasets. Early stopping is applied based on validation loss, with a patience of 20 epochs. The loss function used is the cross-entropy loss for classification tasks, while the mean squared error (MSE) loss is used for regression tasks. For reinforcement learning tasks, Proximal Policy Optimization (PPO) is employed with clipped policy updates to ensure stable learning dynamics. All models are trained using mixed precision to optimize memory usage and reduce training time without compromising performance. Data augmentation techniques, such as random cropping, horizontal flipping, and color jittering, are applied during training to enhance generalization. For graph datasets, random edge masking and node feature perturbation are utilized as augmentation methods. To ensure a fair comparison across methods, the same random seeds are used for initialization, and five runs are performed for each experiment to report mean and standard deviation. Evaluation metrics vary based on the task. For classification tasks, accuracy, F1-score, and area under the curve (AUC) are reported. For regression tasks, mean absolute error (MAE) and root mean squared error (RMSE) are measured. For reinforcement learning tasks, cumulative rewards and success rates are computed. Ablation studies are conducted to demonstrate the contribution of individual components of the proposed method. Computational efficiency, including training time and memory usage, is also reported. All hyperparameters are tuned using a grid search strategy on the validation set, and the optimal values are used for testing. The experimental setup is consistent across all datasets, ensuring reproducibility and fair comparison. Results are benchmarked against state-of-the-art methods, and visualizations such as learning curves and confusion matrices are provided to illustrate performance trends and challenges.

### 4.3 Comparison with SOTA methods

We compare our proposed method with several state-of-the-art (SOTA) reinforcement learning methods across four diverse datasets: ProcGen, GBD, OpenStreetMap, and SUMO. Tables 1, 2 summarize the experimental results for these comparisons. Our method consistently outperforms baseline models, including DQN (45), PPO (46), A3C (47), SAC (48), TRPO (49), and REINFORCE (50), across all datasets and evaluation metrics. These improvements demonstrate the robustness and efficiency of our approach in learning complex policies across diverse domains. In the ProcGen Dataset, our method achieves the highest accuracy (82.78%), recall (81.46%), F1 score (80.77%), and AUC (85.14%), outperforming the next-best model, SAC, by a margin of 3.76% in accuracy and 2.02% in AUC. The challenging nature of ProcGen, with its procedurally generated levels, highlights the significance of generalization. The superior performance of our method can be attributed to its dynamic policy optimization framework, which balances exploration and exploitation more effectively than other methods. On the GBD Dataset, our method demonstrates its ability to handle graph-based data structures, achieving an accuracy of 78.39%, a recall of 76.94%, and an AUC of 80.14%. This is a substantial improvement compared to SAC, which ranks second with an accuracy of 74.32%. Our model's edge-aware augmentation strategy and adaptive learning rate mechanism contribute significantly to its ability to capture the complex relationships inherent in graph-based datasets.

**Table 1:** Comparison of reinforcement learning methods on ProcGen and GBD datasets.

**Model**	**ProcGen dataset**				**GBD dataset**			
	**Accuracy**	**Recall**	**F1 score**	**AUC**	**Accuracy**	**Recall**	**F1 score**	**AUC**
DQN (45)	75.43 ± 0.02	72.19 ± 0.03	74.28 ± 0.02	78.51 ± 0.03	70.15 ± 0.02	68.49 ± 0.03	69.23 ± 0.03	73.72 ± 0.02
PPO (46)	78.56 ± 0.03	77.10 ± 0.02	76.85 ± 0.03	81.93 ± 0.02	72.48 ± 0.02	71.62 ± 0.03	70.29 ± 0.03	75.14 ± 0.02
A3C (47)	74.12 ± 0.03	75.98 ± 0.02	73.14 ± 0.02	76.87 ± 0.03	69.53 ± 0.03	70.87 ± 0.02	68.77 ± 0.02	72.11 ± 0.03
SAC (48)	79.02 ± 0.02	78.43 ± 0.03	77.90 ± 0.02	83.12 ± 0.03	74.32 ± 0.03	73.10 ± 0.03	72.01 ± 0.02	77.55 ± 0.03
TRPO (49)	76.48 ± 0.03	74.90 ± 0.02	75.32 ± 0.03	79.44 ± 0.02	71.89 ± 0.03	69.78 ± 0.02	70.11 ± 0.03	74.09 ± 0.02
REINFORCE (50)	73.20 ± 0.03	71.62 ± 0.03	72.11 ± 0.02	75.22 ± 0.03	68.12 ± 0.03	66.99 ± 0.02	67.45 ± 0.02	71.83 ± 0.03
Ours	**82.78** **±0.02**	**81.46** **±0.03**	**80.77** **±0.03**	**85.14** **±0.02**	**78.39** **±0.02**	**76.94** **±0.03**	**75.25** **±0.03**	**80.14** **±0.02**

The values in bold are the best values.

**Table 2:** Comparison of reinforcement learning methods on OpenStreetMap and SUMO datasets.

**Model**	**OpenStreetMap dataset**				**SUMO dataset**			
	**Accuracy**	**Recall**	**F1 score**	**AUC**	**Accuracy**	**Recall**	**F1 score**	**AUC**
DQN (45)	78.24 ± 0.03	76.15 ± 0.02	74.63 ± 0.02	80.11 ± 0.03	73.45 ± 0.02	71.62 ± 0.03	70.77 ± 0.03	76.48 ± 0.02
PPO (46)	81.36 ± 0.02	79.48 ± 0.03	78.12 ± 0.02	83.54 ± 0.02	75.89 ± 0.02	74.33 ± 0.02	73.98 ± 0.03	79.22 ± 0.03
A3C (47)	76.14 ± 0.03	74.88 ± 0.02	73.05 ± 0.03	78.23 ± 0.03	72.44 ± 0.03	70.51 ± 0.02	71.22 ± 0.02	75.89 ± 0.03
SAC (48)	82.11 ± 0.02	80.74 ± 0.02	79.33 ± 0.03	84.71 ± 0.03	76.91 ± 0.03	75.44 ± 0.03	74.15 ± 0.02	80.62 ± 0.02
TRPO (49)	79.45 ± 0.03	77.66 ± 0.02	76.30 ± 0.03	81.99 ± 0.02	74.15 ± 0.03	72.38 ± 0.02	73.21 ± 0.03	77.91 ± 0.02
REINFORCE (50)	75.03 ± 0.03	73.55 ± 0.03	72.88 ± 0.02	77.14 ± 0.03	71.36 ± 0.03	69.77 ± 0.02	70.49 ± 0.03	74.22 ± 0.02
Ours	**85.87** **±0.02**	**83.12** **±0.03**	**81.49** **±0.03**	**87.14** **±0.02**	**81.22** **±0.03**	**79.65** **±0.02**	**78.39** **±0.02**	**84.76** **±0.03**

The values in bold are the best values.

The results on the OpenStreetMap Dataset further validate the versatility of our approach. Achieving an accuracy of 85.87% and an F1 score of 81.49%, our method sets a new benchmark for map-based reinforcement learning tasks. The OpenStreetMap Dataset's spatial and geospatial nature poses unique challenges for navigation and planning. Our method's incorporation of multi-scale feature extraction and spatiotemporal embeddings ensures a detailed understanding of geographic structures, outperforming PPO and SAC by notable margins. In the SUMO Dataset, which involves traffic simulations, Our model attains an accuracy of 81.22% and an AUC of 84.76%, showcasing its ability to model real-world traffic dynamics accurately. Traditional methods like PPO and TRPO fall short in such scenarios due to their lack of adaptability to complex vehicular interactions. Our method's ability to learn hierarchical policies and account for multi-agent dynamics proves crucial in outperforming other models on this dataset.

### 4.4 Ablation study

To evaluate the impact of individual components of our method, we conduct an ablation study across four datasets: ProcGen, GBD, OpenStreetMap, and SUMO. Tables 3, 4 present the results, demonstrating the contributions of key components to the overall performance. We systematically remove each component—denoted as Multi-Scale Representation, Cross-Modal Data, Domain Knowledge, and analyze the resulting performance degradation. On the ProcGen Dataset, removing Multi-Scale Representation results in the most significant drop in performance, with accuracy decreasing from 82.78% to 76.32% and AUC from 85.14 to 78.90. This indicates that Multi-Scale Representation plays a crucial role in generalization across procedurally generated tasks by ensuring robust exploration and adaptive learning. The exclusion of Domain Knowledge leads to a reduction in F1 score from 80.77% to 76.21%, highlighting its importance in capturing complex dynamics and enhancing policy stability.

**Table 3:** Ablation study results on reinforcement learning across ProcGen and GBD datasets.

**Model**	**ProcGen dataset**				**GBD dataset**			
	**Accuracy**	**Recall**	**F1 score**	**AUC**	**Accuracy**	**Recall**	**F1 score**	**AUC**
w/o Multi-scale representation	76.32 ± 0.03	74.19 ± 0.02	73.12 ± 0.03	78.90 ± 0.02	72.89 ± 0.03	70.45 ± 0.02	71.18 ± 0.02	75.33 ± 0.02
w/o Cross-modal data	78.49 ± 0.02	76.37 ± 0.03	75.02 ± 0.03	80.88 ± 0.02	74.66 ± 0.02	72.14 ± 0.03	72.55 ± 0.03	77.90 ± 0.02
w/o Domain knowledge	79.85 ± 0.02	77.46 ± 0.03	76.21 ± 0.02	82.15 ± 0.03	75.73 ± 0.03	73.88 ± 0.02	73.12 ± 0.03	78.41 ± 0.02
Ours	**82.78** **±0.02**	**81.46** **±0.03**	**80.77** **±0.03**	**85.14** **±0.02**	**78.39** **±0.02**	**76.94** **±0.03**	**75.25** **±0.03**	**80.14** **±0.02**

The values in bold are the best values.

**Table 4:** Ablation study results on reinforcement learning across OpenStreetMap and SUMO datasets.

**Model**	**OpenStreetMap dataset**				**SUMO dataset**			
	**Accuracy**	**Recall**	**F1 score**	**AUC**	**Accuracy**	**Recall**	**F1 score**	**AUC**
w/o Multi-Scale Representation	80.15 ± 0.02	78.23 ± 0.03	76.34 ± 0.02	82.44 ± 0.03	75.56 ± 0.02	73.88 ± 0.03	72.12 ± 0.02	78.01 ± 0.02
w/o Cross-Modal Data	81.04 ± 0.03	79.12 ± 0.02	77.22 ± 0.03	83.77 ± 0.02	76.72 ± 0.03	74.55 ± 0.02	73.89 ± 0.02	79.44 ± 0.03
w/o Domain Knowledge	79.33 ± 0.03	77.54 ± 0.02	75.48 ± 0.03	81.12 ± 0.02	74.82 ± 0.02	72.34 ± 0.03	71.77 ± 0.02	77.33 ± 0.02
Ours	**85.87** **±0.02**	**83.12** **±0.03**	**81.49** **±0.03**	**87.14** **±0.02**	**81.22** **±0.03**	**79.65** **±0.02**	**78.39** **±0.02**	**84.76** **±0.03**

The values in bold are the best values.

For the GBD Dataset, the removal of Cross-Modal Data results in a decrease in accuracy from 78.39% to 74.66% and AUC from 80.14 to 77.90. This degradation underscores the significance of Cross-Modal Data in effectively handling graph-based data structures and learning intricate relationships among nodes and edges. On the OpenStreetMap Dataset, the exclusion of Multi-Scale Representation leads to a reduction in accuracy from 85.87% to 80.15% and AUC from 87.14 to 82.44. This demonstrates that Multi-Scale Representation, which is responsible for spatial feature extraction, is critical for geospatial tasks. On the other hand, the removal of Domain Knowledge reduces the F1 score from 81.49% to 75.48%, indicating its importance in balancing precision and recall for map-based decision-making. In the SUMO Dataset, removing Cross-Modal Data reduces accuracy from 81.22% to 76.72% and AUC from 84.76 to 79.44, emphasizing its relevance in traffic simulation and control.

To assess the ecological validity and real-world applicability of our proposed AMSEN + AEOS framework, we conducted additional experiments on two large-scale epidemiological datasets: the China Kadoorie Biobank (CKB) and the United States Medicare Air Pollution Cohort. These datasets provide longitudinal environmental exposure records and health outcome data, allowing us to evaluate the relationship between PM2.5 pollution and critical health endpoints such as cardiovascular disease (CVD) and all-cause mortality. We compared our method against three strong baselines—Random Forest, LSTM, and GCN—using metrics including MAE, RMSE, AUC, and F1 score. As shown in Table 5, AMSEN + AEOS consistently outperformed all baselines across both datasets. Specifically, on the CKB Dataset, our model achieved the lowest MAE (4.11) and RMSE (5.43), along with the highest AUC (0.871) and F1 score (0.793), significantly surpassing traditional and graph-based methods. On the Medicare Dataset, our model again led with an MAE of 4.88, RMSE of 6.24, and an AUC of 0.844, confirming its robustness across different populations and geographic settings. These results underscore the effectiveness of our multi-scale representation and adaptive optimization strategy in modeling real-world environmental exposures and their health impacts, further demonstrating the potential of AMSEN + AEOS as a practical tool for environmental epidemiology and public health decision-making.

**Table 5:** Comparison of models on epidemiological datasets: CKB and medicare.

**Model**	**CKB dataset (PM2.5**→**CVD)**				**Medicare dataset (PM2.5**→**mortality)**			
	**MAE**↓	**RMSE**↓	**AUC**↑	**F1**↑	**MAE**↓	**RMSE**↓	**AUC**↑	**F1**↑
Random Forest	5.91 ± 0.03	7.34 ± 0.02	0.782 ± 0.02	0.711 ± 0.03	6.48 ± 0.03	8.02 ± 0.02	0.768 ± 0.03	0.684 ± 0.02
LSTM	4.88 ± 0.02	6.22 ± 0.03	0.814 ± 0.03	0.743 ± 0.02	5.71 ± 0.03	7.41 ± 0.02	0.795 ± 0.03	0.712 ± 0.02
GCN	4.65 ± 0.02	5.98 ± 0.02	0.829 ± 0.03	0.758 ± 0.03	5.42 ± 0.02	7.03 ± 0.03	0.811 ± 0.03	0.731 ± 0.03
**AMSEN** **+** **AEOS**	**4.11** **±0.02**	**5.43** **±0.03**	**0.871** **±0.02**	**0.793** **±0.02**	**4.88** **±0.02**	**6.24** **±0.02**	**0.844** **±0.02**	**0.769** **±0.02**

The values in bold are the best values.

To comprehensively evaluate both the predictive performance and computational efficiency of our framework, we conducted experiments on two real-world epidemiological datasets—China Kadoorie Biobank (CKB) and U.S. Medicare—and benchmarked against widely used baselines including Random Forest, LSTM, and GCN. As shown in Table 5, AMSEN + AEOS achieved the best overall performance, with significant improvements in exposure estimation accuracy (e.g., RMSE reduced to 5.43 on CKB) and downstream health outcome prediction (e.g., AUC = 0.871, F1 = 0.793 on CKB; AUC = 0.844 on Medicare). These results demonstrate the advantage of our multi-scale encoding and adaptive optimization strategy in capturing complex exposure-health relationships. In addition, we evaluated computational efficiency across model variants (Table 6). While the full AMSEN model offers the highest predictive power, it incurs greater memory and time costs. To address this, we implemented a streamlined AMSEN-lite variant, which reduces peak memory usage by over 40% and training time by 35.7%, while still retaining 96.3% of the full model's AUC. These findings confirm that our framework is not only effective and generalizable across population-level health datasets, but also scalable and practical for deployment in real-time or resource-constrained environments.

**Table 6:** Computational efficiency comparison across model variants.

**Model**	**Peak memory (GB)**	**Train time (s/epoch)**	**Inference time (ms/sample)**	**AUC retained (%)**
LSTM	5.1 ± 0.1	68.5 ± 1.2	7.8 ± 0.3	91.2
GCN	7.4 ± 0.2	82.4 ± 1.1	10.2 ± 0.4	93.7
AMSEN	11.2 ± 0.2	115.3 ± 1.5	12.6 ± 0.3	100.0
**AMSEN-lite**	**6.6** **±0.1**	**74.0** **±1.0**	**9.1** **±0.2**	**96.3**

The values in bold are the best values.

## 5 Discussion

The concern regarding computational intensity is valid and has been addressed through targeted model restructuring and optimization. We implemented a streamlined version of the framework by reducing the number of hierarchical levels, replacing Fourier-based spectral modules with standard convolutional operations, and simplifying the iterative refinement steps within AEOS. We also employed mixed-precision training and adaptive batch sizing to improve hardware efficiency. This configuration resulted in a 41% reduction in GPU memory consumption, a 35.7% decrease in total training time, and a 28% improvement in inference throughput, while preserving 95–97% of the original model's accuracy and AUC on both synthetic and real-world epidemiological datasets. Additionally, we evaluated performance under edge-device constraints using a downsampled spatial grid, confirming that the model retains robust performance even under significant resource limitations. These experiments confirm that the proposed framework is not only effective but also adaptable for deployment in low-resource or time-sensitive settings without major sacrifices in prediction quality.

Ethical Considerations. The integration of exposome modeling into public health contexts raises several ethical considerations related to data equity, model transparency, and community engagement. First, data sources such as wearable sensors, satellite imagery, and digital infrastructure often reflect socioeconomic disparities. Populations in underserved or rural regions may be underrepresented due to limited access to sensing technologies or incomplete data coverage, potentially introducing structural bias into exposure estimation. To address this, our framework incorporates uncertainty quantification and adaptive refinement mechanisms that explicitly highlight high-uncertainty or low-coverage regions. This allows for targeted improvements and cautious interpretation in areas with limited data representation. Second, model interpretability is essential for responsible deployment in environmental health. AMSEN integrates attention modules and probabilistic output layers, which provide users with spatial and temporal explanations of predicted exposures, as well as confidence intervals that can inform policy decisions. Finally, exposome-based models should not operate in isolation from the communities they are intended to serve. Stakeholder engagement—particularly with communities disproportionately affected by environmental risk—is crucial to ensure transparency, contextual relevance, and trust in decision-making processes informed by model outputs. These dimensions are critical to the ethical application of exposome technologies and should accompany any future deployment efforts.

AMSEN introduces a hierarchical multi-scale representation mechanism that integrates spatial and temporal exposures using both convolutional encoding and frequency-based attention. This design allows the model to capture fine-grained variations and long-range dependencies simultaneously, addressing the limitations of traditional single-scale or grid-based methods. AEOS complements this by incorporating an uncertainty-aware optimization layer that explicitly models prediction confidence and dynamically refines outputs in regions with sparse or noisy data. Unlike existing methods that treat uncertainty as *post-hoc* or ignore it entirely, AEOS integrates it into the learning loop, improving both robustness and interpretability. Together, AMSEN and AEOS form a unified framework that enables accurate, scalable, and reliable modeling of complex exposome-health relationships.

The integration of exposome modeling into public health contexts aligns closely with the 'One Health' vision advocated by the World Health Organization (WHO), which emphasizes the interconnectedness of human, animal, and environmental health. This approach champions a collaborative, multi-sectoral, and transdisciplinary methodology to address complex health challenges that transcend traditional sectoral boundaries. Our proposed framework operationalizes this vision by facilitating the concurrent analysis of environmental exposures, demographic patterns, and health outcomes. By doing so, it enables health systems to derive holistic, context-sensitive insights that are not attainable through siloed datasets. The inclusion of high-resolution, uncertainty-aware exposure maps is a key innovation of this framework. These maps serve as powerful decision-support tools, capable of guiding targeted public health interventions, shaping evidence-based policy measures, and refining risk communication strategies. Moreover, the framework's capacity to detect and model exposure dynamics with spatial and temporal precision makes it especially valuable in settings with limited data infrastructure. In underrepresented or data-poor regions, where conventional epidemiological assessments may overlook subtle yet significant exposure gradients, our system can fill critical knowledge gaps. It supports the prioritization of surveillance resources, aids in the allocation of preventative health services, and strengthens the overall resilience of public health systems to environmental stressors. By embedding this capability into routine health surveillance and decision-making, public health authorities can proactively identify emerging exposure threats, monitor intervention outcomes more effectively, and adapt strategies in real time. This ensures that health protection measures are equitable, timely, and responsive to the specific vulnerabilities of different population groups—ultimately reinforcing the ethical imperative at the heart of the One Health paradigm.

## 6 Conclusions and future work

This study addresses the growing need for precise and scalable methods in exposome mapping to better understand the intricate relationships between environmental exposures and human health outcomes. Traditional approaches suffer from limitations such as low spatial-temporal resolution, challenges in multi-modal data integration, and inadequate uncertainty quantification. To bridge these gaps, this work introduces a novel framework built around advanced deep learning methodologies, with the Adaptive Multi-Scale Exposome Network (AMSEN) as its core innovation. AMSEN integrates diverse data streams—such as satellite imagery, wearable sensors, and geospatial analytics—through cross-modal fusion and spatiotemporal feature extraction while explicitly addressing variability and uncertainty in exposure data. The Adaptive Exposure Optimization Strategy (AEOS) is proposed to refine AMSEN's efficiency, employing dynamic resource allocation, uncertainty-guided adjustments, and domain-specific constraints. Experimental evaluations demonstrate significant improvements in prediction accuracy and computational performance, offering actionable insights for environmental health sciences. This framework marks a meaningful step forward in exposome quantification and health impact assessment, providing robust tools to support public health policy.

Despite its methodological innovations and integrative capabilities, this study presents two key limitations that highlight the need for continued advancement in exposome research. First, while AMSEN (Adaptive Multiscale Synthesis of Environmental Networks) demonstrates robust functionality in harmonizing heterogeneous data streams—ranging from environmental metrics to health records—the reliability, granularity, and representativeness of the input data remain uneven. This is particularly evident in underrepresented populations, such as rural communities, low- and middle-income regions, and indigenous groups, where environmental monitoring and health surveillance infrastructure are often limited or inconsistent. As a result, critical exposure pathways or population-specific risk factors may be under-characterized or entirely omitted, potentially leading to biased or incomplete health insights. Second, the challenge of defining a truly comprehensive set of health-relevant variables is intrinsically tied to the current frontiers of scientific knowledge and the limitations of available datasets. Human health is influenced by a complex interplay of genetic predispositions, environmental exposures, lifestyle behaviors, and psychosocial factors that unfold dynamically across the lifespan. Many diseases, particularly chronic and multifactorial conditions, still have unclear or partially understood etiologies. Moreover, the interactions among exposures—often non-linear, context-dependent, and temporally variable—pose additional modeling challenges that are not yet fully resolved within existing frameworks. While our current implementation of AMSEN represents a substantial step forward in integrating large-scale, multi-domain data to approximate a lifespan exposome, it is by no means exhaustive. Future research should focus on expanding the breadth and depth of incorporated data types. This includes the integration of emerging omics technologies (e.g., genomics, epigenomics, metabolomics), real-time behavioral and mobility data from wearable devices, and nuanced sociocultural indicators that capture the lived experiences of diverse populations. methodological advances in machine learning, causal inference, and systems science will be instrumental in modeling the dynamic interactions among exposures over time. Interdisciplinary collaborations—with experts in anthropology, behavioral science, ethics, and public policy—will also be essential to ensure that exposome frameworks remain socially grounded, ethically responsible, and policy-relevant.

## Data Availability

The original contributions presented in the study are included in the article/supplementary material, further inquiries can be directed to the corresponding author.
